# Protecting the Safe Water Chain in Refugee Camps: An Exploratory Study of Water Handling Practices, Chlorine Decay, and Household Water Safety in South Sudan, Jordan, and Rwanda

**DOI:** 10.4269/ajtmh.24-0221

**Published:** 2024-12-03

**Authors:** Syed Imran Ali, Michael De Santi, Georges Monette, Usman T. Khan, Jean-François Fesselet, James Orbinski

**Affiliations:** ^1^ Dahdaleh Institute for Global Health Research, York University, Toronto, Ontario, Canada;; ^2^ Public Health Department, Médecins Sans Frontières, Amsterdam, The Netherlands;; ^3^ Blum Center for Developing Economies, University of California, Berkeley, California;; ^4^ Lassonde School of Engineering, York University, Toronto, Ontario, Canada;; ^5^ Department of Mathematics and Statistics, York University, Toronto, Ontario, Canada

## Abstract

In refugee and internally displaced person settlements, hygienic water handling and free residual chlorine (FRC) are crucial for protecting water against recontamination after distribution up to the household point-of-consumption. We conducted a secondary analysis of water quality and water handling data collected in refugee camps in South Sudan, Jordan, and Rwanda using statistical and process-based modeling to explore how water handling practices affect FRC decay and household FRC outcomes. The two practices that consistently produced a significant effect on FRC decay and household FRC were storing water in direct sunlight and transferring water between containers during household storage. Samples stored in direct sunlight had 0.22–0.31 mg/L lower household FRC and had FRC decay rates between 2 and 3.7 times higher than samples stored in the shade, and samples that were transferred between containers had 0.031–0.51 mg/L lower household FRC and decay rates 1.65–3 times higher than non-transferred samples in sites in which the effect was significant, suggesting that humanitarian responders should aim to provide additional water storage containers to prevent water transferring in households and encourage water-users not to store water in direct sunlight. By contrast, the effect of the three recommended hygienic water handling behaviors (clean, covered containers and drawing by tap or pouring) was mixed or inconclusive. These inconclusive results were likely due to imbalanced or unreliable approaches to gathering the data, and we recommend that hygienic water handling practices that mechanistically provide a physical barrier against recontamination should always be promoted in humanitarian settings.

## INTRODUCTION

Waterborne illnesses, such as cholera, hepatitis E, and shigellosis, are among the leading causes of epidemics in refugee and internally displaced person (IDP) settlements worldwide.^1^ Ensuring that water is protected from pathogenic recontamination and remains safe to drink up to the household point-of-consumption is critical for protecting public health in these settings. In many refugee and IDP settlements, water is not piped to individual households but is instead collected by water users from public distribution points (tapstands), transported to dwellings, and then stored and used over the course of hours or days. Thus, even if water is microbiologically safe when it is collected from the tapstand, it can be recontaminated by user and environmental interactions during this post-distribution phase. Post-distribution recontamination has been identified as a contributing factor in numerous outbreaks of waterborne illnesses in refugee settlements in Kenya,^2^^–^^4^ Malawi,^5^^,^^6^ Sudan,^7^ and South Sudan.^8^^,^^9^

Humanitarian water, sanitation, and hygiene (WASH) teams treat water to remove and inactivate pathogens and ensure that water is acceptable to users. Free residual chlorine (FRC) is used as a secondary disinfectant to provide a chemical barrier against recontamination by inactivating fecal–oral pathogens that may be introduced to water after treatment.^10^ Humanitarian WASH teams also promote hygienic water handling practices (covering containers, drawing water via a tap or pouring, and keeping containers clean) to provide physical barriers against recontamination.^10^ These chemical and physical barriers are essential components of the safe water chain, which is the combination of water treatment, distribution, and storage processes from source to user that prevent contamination and protect public health.^8^^,^^11^^,^^12^

Although water treatment is well understood, there remain gaps in the literature regarding the post-distribution period, particularly in refugee and IDP settings. This a concern because post-distribution recontamination is a contributing factor in many outbreaks^2^^–^^9^; refugees and displaced persons generally have lower access to basic or improved WASH services, even when they obtain water from improved sources^13^; and a lack of WASH services, especially when combined with overcrowding, malnutrition, or lack of access to healthcare, can lead to high morbidity and mortality due to infectious disease.^14^^,^^15^ A comparison of factors associated with diarrhea in refugee and host communities in Gambella, Ethiopia, revealed that refugees were more likely to experience diarrhea than host communities,^16^ despite a study in the same communities finding that refugees had much better source water quality than the host community.^17^ Although these studies did not identify specific differences in causes of recontamination, the magnitude of recontamination was much higher in the refugee settlement,^17^ and diarrhea in refugee settlements was more strongly associated with container covering than in the host community.^16^ Thus, preventing post-distribution contamination is a critical step to preventing waterborne illness outbreaks among refugees, a population that is particularly vulnerable to these illnesses.

Residual chlorination has been demonstrated as the most effective means of preventing pathogenic recontamination during the post-distribution period,^18^^,^^19^ but chlorine decay can eventually leave water vulnerable to recontamination,^5^^,^^8^^,^^20^ so it is important to understand how water handling, as a physical barrier to recontamination, interacts with the chemical barrier provided by residual chlorine. The mechanics of these barriers individually are well understood. Numerous studies from both emergency and stable settings have demonstrated microbiological contamination of water during collection, transport, and storage,^21^^–^^24^ and researchers have investigated the mechanics of contamination and the effects of specific behaviors,^20^^,^^21^^,^^25^^–^^28^ container types (material, aperture size, presence of covers and taps),^5^^,^^18^^,^^19^^,^^29^^,^^30^ and environmental and sociological factors^5^^,^^22^^,^^31^ on post-distribution contamination. Other studies have demonstrated the effectiveness of residual chlorination in preventing recontamination by both fecal indicator organisms and *Vibrio cholerae*, an important waterborne pathogen in humanitarian contexts.^18^^,^^19^^,^^32^ However, aside from one study by Gärtner et al.,^33^ which demonstrated that cleaned and disinfected containers reduced FRC loss in water containers in rural communities in eastern Uganda, there is little evidence on how water handling affects residual chlorine protection, and to the best of our knowledge, there has been no such research in high-risk settings such as refugee and IDP settlements.

This paper presents an exploratory study of how water handling practices affect post-distribution chlorine decay in refugee and IDP settlements. We conducted a secondary analysis of water quality and water handling behavioral data collected from refugee camps in South Sudan, Jordan, and Rwanda^34^ using statistical and process-based models to assess the effect of various water handling practices on point-of-consumption FRC concentrations and post-distribution FRC decay. By better understanding how water handling practices affect residual chlorine protection, our goal is to identify best practices in humanitarian settings that preserve residual chlorine, thereby extending its protective role in the safe water chain during the critical post-distribution period.

## MATERIALS AND METHODS

### Study dataset and site background.

The data used for this secondary analysis come from a multisite investigation of post-distribution chlorine decay in refugee camps in South Sudan, Jordan, and Rwanda.^34^ The dataset consists of water quality and water handling behavioral data collected at these sites between 2013 and 2015. Key site characteristics, including environmental conditions, demographics, and details of WASH service levels, are provided in Table 1. Environmental hygiene was considered poor in both South Sudan and Rwanda but was better in the planned Azraq camp in Jordan. We also treated the two datasets obtained from Jordan as separate because they were collected 9 months apart under different seasonal and population conditions. Further details on these sites can be obtained in the original study.^34^

**Table 1:** Study site characteristics, including environmental conditions, population, and water, sanitation, and hygiene service levels

Country	Site(s)	Ambient Air Temperature (°C)	Population	Water Availability (liters per capita per day)	Water Accessibility (users per tap)	Sanitation Availability (persons per latrine)	Number of Samples in Dataset
South Sudan	Batil	Average: 35.3 (Min: 28.3; Max: 45.7)	37,199	18.9	97*	20	69
	Gendrass		15,810	25.6	88*	14	76
	Jaman		15,670	19.3	84*	19	75
Jordan (Summer 2014)	Azraq	Average:32.7 (Min: 27.1; Max: 43.3)	10,000–12,000	36.9	63	4.5	199
Jordan (Winter/Spring 2015)	Azraq	Average:21.7 (Min: 14.5; Max: 29.3)	20,000	21.6	123*	8.8	140
Rwanda	Kigeme	Average:22.2 (Min: 18.3; Max: 31.0)	18,569	13.6*	135*	33*	134
			Sphere indicator:	>15	≤80	≤20	–

* Fails to meet Sphere indicator^10^

### Data collection.

Water quality data included the following parameters, which are routinely collected in humanitarian response: FRC, total residual chlorine, electrical conductivity, water temperature, turbidity, and pH. Water quality data were collected in a series of water quality surveys that occurred between the tapstand and the household point-of-consumption, with the same unit of water being sampled each time. Water quality data were collected first directly from the tap at the point-of-distribution (tapstand); second from containers immediately after collection; third from containers immediately after water was transported back to the dwelling; and fourth from containers in the dwelling after 4–26 hours of household storage and use. We subdivided the post-distribution period into three distinct phases (A, B, and C) bounded by two water quality surveys: phase A between the tap and collection into containers, phase B between collection and arrival at the dwelling, and phase C between water arriving at the dwelling and the final household measurement. This approach allowed us to explore FRC decay specifically during post-distribution phases B (collection and transport) and C (storage and use) independently to discern if certain factors were more influential during one phase or another.

Water handling practices were documented as self-reported or observed behaviors during two water handling surveys: first during collection at the tapstand and second at the household point-of-consumption. Water handling practices are outlined in Table 2. Container cleanliness, container covering, and drawing method are the three key hygienic water handling practices stipulated in humanitarian guidelines.^10^ We combined the two hygienic water drawing behaviors (i.e., pouring and using a tap) into a single variable. Water storage in direct sunlight was only observed in Jordan and not at other sites; it began to be documented upon observation of the practice in Jordan because it is well-established that sunlight can cause photonic degradation of FRC^35^ and increase FRC decay through increased temperature,^36^ leading to increased volatilization and reactivity. Mixing, transferring, and using water in the home are behavioral indicators of user interactions with water during household storage and use and were documented through self-report to assess whether user interactions affected FRC decay. Water mixing refers to instances in which the original unit of water that was tested at the tapstand had another unit of water mixed into it during household storage, such that the water tested in the final water quality survey was a mixture of two or more different units of water. Supplemental Table 1 includes photographs from the field that illustrate the categories for container cleanliness, covering, drawing method, and storage in direct sunlight.

**Table 2:** Water handling practices documented in the dataset used in this study

Water Handling Practice	Binary Categories	Relevant Phase	Assessment Method
Container cleanliness	Clean, Unclean	B: Collection and transport, C: Storage and use	Visual observation by surveyor
Container covering	Covered, Uncovered	B: Collection and transport, C: Storage and use	Visual spot-check by surveyor
Drawing method	Tap/Pour, Dip	C: Storage and use	Respondent self-report
Storage in the sun or shade	Sun storage, Shade storage	C: Storage and use	Spot-check by surveyor
Water mixing	Mixed, Unmixed	C: Storage and use	Respondent self-report
Water transferring	Transferred, Not transferred	C: Storage and use	Respondent self-report
Water use	Used, Unused	C: Storage and use	Respondent self-report

**Figure 1.**
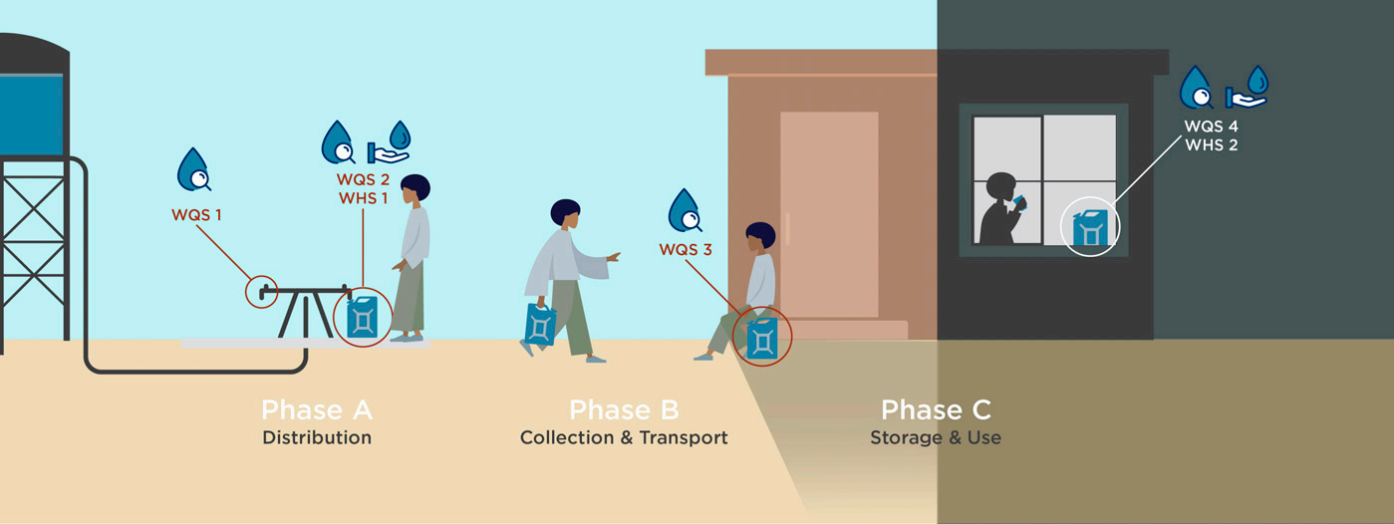
Schematic illustration of data collection methodology. WHS = water handling survey; WQS = water quality survey.

Therefore, each unique sample in the dataset had four water quality surveys and two water handling surveys. Figure 1 schematically illustrates the data collection methodology, including where each survey took place and phases A (distribution), B (collection and transport), and C (storage and use).

Further details on water quality and water handling data collection and sampling design are available in the study reporting the original dataset.^34^

### Analytical approaches.

To assess the influence of water handling practices on residual chlorine protection, we took two approaches—one examining household FRC outcomes and the other examining FRC decay rates.

Both approaches were based, directly or indirectly, on the first-order chlorine decay model, which is an empirical reaction kinetics model. This model assumes that the decay rate is constant over time, to which we fit model parameters using field data. The first-order decay model has been shown to perform comparably to more complex chlorine decay models^37^^,^^38^ and has two key advantages. The first is that with the only free parameter being the decay rate (*k*), first-order models are easily comparable. We can also fit the first-order model to longitudinal observations by using linear regression models. Equation 1 shows the integrated form of the first-order decay equation, where *C_o_* is the initial FRC concentration (mg/L), *C* is the FRC concentration (mg/L) at time *t* (hours), and *k* is the decay rate (hours^−1^).

$$C=C_{o}e^{-kt}$$ (1)

The first-order decay equation can be expressed as a linear regression model by taking the natural logarithm of Equation 1. Thus, in Equation 2, the decay rate *k* becomes the regression coefficient, with *t* as the endogenous variable and ln(*C_0_*) as the intercept.

$$\ln\;\left(C\right)=\ln\left(C_{0}\right)-kt$$ (2)

#### Approach 1: Effect of water handling practices on household FRC outcomes.

The first approach sought to assess whether water handling practices affect household FRC. We used a multiple linear regression model with the natural logarithm of household FRC as the target variable. We included an interaction term between the natural logarithm of FRC measured at the tapstand and the elapsed time between the tapstand and household measurements with a regression coefficient β to control for the effect of chlorine decay on household FRC, and then we introduced a binary variable, x, representing the water handling behavior being tested (e.g., clean versus unclean containers) with the regression coefficient α, as shown in Equation 3.

$$\ln\;\left(C\right)=\beta *t\ln\;\left(C_{0}\right)+\alpha *x$$ (3)

The effect of the water handling behavior is reflected in the coefficient α. A positive α indicates that the behavior (e.g., covering a container) is associated with higher average FRC concentrations at the end of household storage than the inverse of that behavior (e.g., not covering) and thus indicates that the behavior helps preserve the safe water chain. We evaluated the significance of effects using the *P*-value of the regression coefficient, with effects considered significant at a *P*-value of 0.05 or lower.

The advantage of this analytical approach is that the model directly quantifies the effect of water handling practices on the target outcome of household FRC, which is the variable that best reflects whether water is protected against pathogenic recontamination up to the end of the household storage and use phase. In addition, the multiple linear regression approach allows all relevant water handling practices to be included in the model simultaneously so that the effect of each can be evaluated while controlling for the effect of others. However, one limitation of this approach is that the multiple linear regression model can be affected by multicollinearity if two or more behaviors are correlated with one another. Multicollinearity reduces the accuracy of the effect estimated by the model and can weaken statistical inference. In preliminary data analysis, we found that most households that collected water in clean or covered containers also stored water in clean or covered containers, so we only evaluated container cleanliness and covering during household storage and use (phase C) and dropped these variables during collection and transport (phase B).

#### Approach 2: Effect of water handling practices on FRC decay rate.

The second approach explored the effect of water handling practices on the FRC decay process itself by developing a linear mixed effects (LME) model. The linear model is based on Equation 2, with* k* as the slope and $ln(C_{0})$ as the intercept. In this model, FRC measurements from all water quality surveys for an individual sample are used to find the decay rate in each phase. The LME models contain two types of effects: fixed and random effects. The fixed effects components of the LME model behave essentially the same as a conventional linear regression, whereas random effects allow for and quantify variations in model parameters. Fixed effects in the model include decay rates for all three phases (A, B, and C) grouped by site and an additional site-specific intercept. Random effects in the model are used to quantify variation in the decay rate between different samples. This is a key advantage of the LME modeling approach, given the considerable variability observed in post-distribution FRC decay in real-world settings.^34^^,^^39^ With these variable decay rates for each sample, we could obtain a separate estimate of the decay rate,* k*, for each water handling practice. For example, the LME model produced separate estimates of the decay rate,* k*, during phase B (collection and transport) for covered containers versus uncovered containers at each site. If the decay rate was lower for one binary category (e.g., covered container) as opposed to the opposite binary category (e.g., uncovered container), this indicated that container covering reduced the rate of FRC decay and, therefore, helped preserve the safe water chain. We evaluated the statistical significance of the difference in decay rates for each binary using a Wald test and considered differences with a* P*-value of 0.05 or less to be statistically significant. Although an advantage of this approach is that it assesses the effect that a water handling practice has directly on the FRC decay process itself, a limitation in the present study was that there were not enough data in the dataset to split* k* for more than one water handling practice variable at a time, and thus there may be some confounding between water handling practices.

## RESULTS

### Summary of water handling practices.

**Figure 2.**
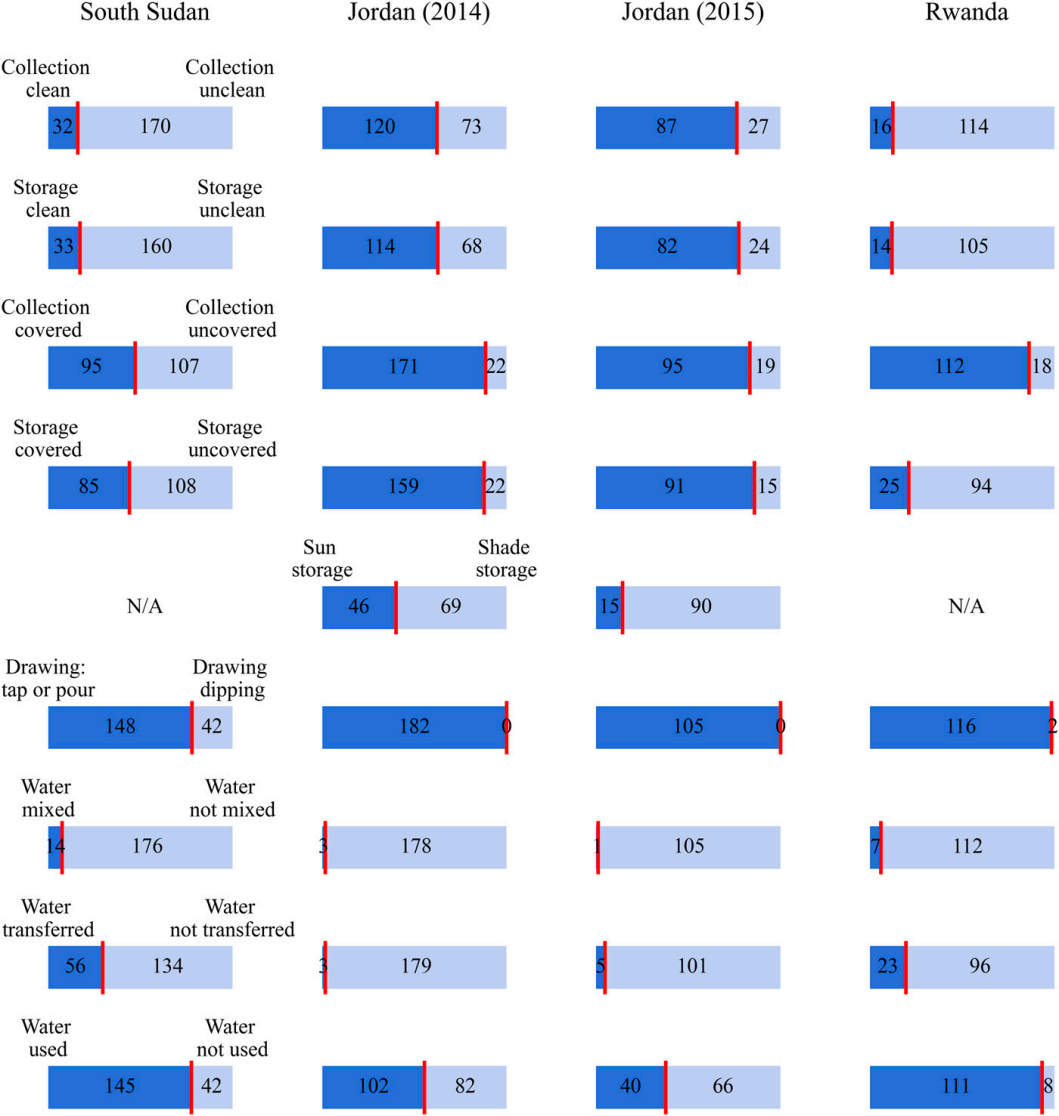
Summary of water handling practices at each site. Rows from top to bottom show collection container cleanliness, storage container cleanliness, covering of collection containers, covering of storage containers, storage in direct sunlight or shade, water drawing method, and finally, whether water was mixed, transferred between containers, or used in households.

The distribution of water handling practices during the four field studies was unique to each site (Figure 2). For example, data from Jordan 2014 and Jordan 2015 show that collection and storage containers were predominantly clean and covered, whereas in South Sudan and Rwanda, water was predominantly collected in unclean containers and stored in uncovered containers. Furthermore, some practices were only observed at some sites—for instance, the storage of water in direct sunlight was only observed in Jordan, and drawing water by dipping was only observed in South Sudan and Rwanda (albeit only twice in the latter).

The Sphere Guidelines recommend that water should be stored in clean, covered containers and drawn by pouring or a tap.^10^ During household storage and use (phase C), all water users in Rwanda and Jordan and 93% (178 out of 191) of water users in South Sudan followed at least one of these hygienic water handling practices. In Jordan, 88% (159 of 181) of sampled households in 2014 and 86% (91 of 106) in 2015 followed all three of these practices, whereas only 44% (85 of 193) of sampled households in South Sudan and 21% (25 of 119) in Rwanda followed all three recommended practices. This shows that, overall, water users adhered to at least some of the recommended hygienic water handling practices in South Sudan and Rwanda, whereas in Jordan, there was good adherence to all recommended practices.

### Effect of water handling practices on household FRC and FRC decay.

As outlined above, we took two approaches to explore the effect of water handling practices on household FRC outcomes and FRC decay rates, the results of which are summarized respectively in Tables 3 and 4. These tables report modeling outputs, including regression coefficients (i.e., α for Approach 1 and *k* for Approach 2) for each water handling practice variable, as well as associated *P*-values. Bolded values denote variables that were significant at the *P* <0.05 threshold for the given model. Estimated *k*-values and their confidence intervals from the LME model in Approach 2 are presented in Supplemental Figures 1 and 2. In the following sections, we outline the findings for each water handling practice on household FRC outcomes and FRC decay from our analysis.

**Table 3:** Outputs from multiple linear regression model on the effects of water handling practices on household free residual chlorine outcomes (Approach 1)

Water Handling Practice	South Sudan		Jordan (2014)		Jordan (2015)		Rwanda	
	α	*P*	α	*P*	α	*P*	α	*P*
Storage container clean	**−0.20**	**0.047**	0.061	0.32	−0.052	0.606	**0.18**	**0.0010**
Storage container covered	0.01	0.84	0.042	0.60	0.0073	0.316	**−0.14**	**0.0014**
Storing water in the sun	n/a	n/a	**−0.22**	**0.00037**	**−0.31**	**<1 × 10^−5^**	n/a	n/a
Drawing water by pouring or tap	0.020	0.81	n/a	n/a	n/a	n/a	−0.17	0.17
Mixing water during storage	**0.37**	**0.0068**	0.079	0.68	0.26	0.20	0.068	0.43
Transferring water during storage	−0.031	0.68	**−0.51**	**0.030**	−0.18	0.055	**−0.12**	**0.0036**
Using water during storage	−0.00077	0.99	0.0029	0.96	0.0050	0.89	**−0.22**	**0.0012**

The table reports the regression coefficient, α, and associated *P*-value for each water handling practice variable. A positive α indicates that the practice is associated with higher household free residual chlorine than the inverse practice and thus helps preserve the safe water chain. Bolded values denote variables that were significant at the *P* < 0.05 threshold.

**Table 4:** Outputs from linear mixed effects model on the effects of water handling practices on free residual chlorine decay rates (Approach 2)

Water Handling Practice	Phase	South Sudan		Jordan (2014)		Jordan (2015)		Rwanda		Overall
		*k*	*P*	*k*	*P*	*k*	*P*	*k*	*P*	*P*
Collection container is clean	B	−0.397	0.657	−0.426	0.607	−0.448	0.340	−0.282	0.669	0.817
Collection container is unclean		−0.481		−0.492		−0.295		−0.427		
Storage container is clean	C	−0.103	0.532	−0.092	0.695	−0.039	0.683	−0.022	0.248	0.727
Storage container is unclean		−0.116		−0.977		−0.033		−0.037		
Collection container is covered	B	−0.434	0.653	−0.490	0.140	**−0.471**	**0.041**	**−0.054**	**0.040**	**0.030**
Collection container is uncovered		−0.499		−0.221		**−0.088**		**−0.482**		
Storage container is covered	C	−0.109	0.451	−0.089	0.076	−0.035	0.157	−0.035	0.955	0.220
Storage container is uncovered		−0.120		−0.130		−0.059		−0.035		
Water is stored in direct sunlight	C	N/A	–	**−0.222**	**<1 × 10^−5^**	**−0.058**	**0.030**	N/A	–	**<1 × 10^−5^**
Water is stored in the shade		N/A		**−0.060**		**−0.029**		N/A		
Drawing by tap or pouring	C	−0.113	0.507	N/A	–	N/A	–	**−0.033**	**0.042**	0.101
Drawing by dipping a vessel		−0.127		N/A		N/A		**−0.066**		
Water was mixed in the dwelling	C	−0.109	0.842	−0.074	0.712	−0.021	0.775	**−0.086**	**0.044**	0.363
Water was not mixed in the dwelling		−0.115		−0.095		−0.038		**−0.034**		
Water was transferred between vessels during storage	C	**−0.159**	**1.2 × 10^−3^**	−0.043	0.362	**−0.104**	**0.009**	**−0.056**	**0.004**	**<1 × 10^−5^**
Water was stored in the same vessel it was collected in		**−0.096**		−0.095		**−0.034**		**−0.029**		
Water was used during the storage period	C	**−0.128**	**0.001**	−0.102	0.207	−0.036	0.843	−0.036	0.261	**0.011**
Water was not used during the storage period		**−0.069**		−0.084		−0.039		−0.015		

The table reports the phase-specific decay rates, *k*, and associated *P*-values. When comparing the two *k*-values for each water handling practice binary, the higher *k* (i.e., less negative) indicates slower free residual chlorine decay; thus, the practice helps preserve the safe water chain. Bolded values denote variables that were significant at the *P* < 0.05 threshold.

#### Container cleanliness.

Table 3 shows that the observed effect of storing water in clean containers on household FRC was mixed (statistically significant in South Sudan and Rwanda but with effects having the opposite direction) or inconclusive (not statistically significant in Jordan 2014 and Jordan 2015). Collection containers were not considered in Approach 1 because of the multicollinearity between collection and storage containers, as described in the description of Approach 1. Table 4 shows that collecting and storing water in clean containers versus unclean containers was not observed to have a statistically significant effect. Based on the analysis of these data, we did not find statistically significant evidence of the presumed protective effect of container cleanliness on household FRC or FRC decay rates.

#### Container covering.

Table 3 shows that a statistically significant protective effect on household FRC from storing water in covered containers was not observed in South Sudan, Jordan 2014, and Jordan 2015; however, a statistically significant but counter-intuitive effect was observed in Rwanda (collection containers were not considered because of multicollinearity). Table 4 shows that collecting water in covered containers had a statistically significant effect on FRC decay rates overall, but the effect was mixed and worked in opposing directions at the two sites where it was significant (Jordan 2015 and Rwanda). Storing water in covered containers was not observed to have a statistically significant effect on FRC decay rates, either overall or at any specific site. Based on the analysis of these data, we found mixed and inconclusive evidence on the presumed protective effect of container covering.

#### Storage in direct sunlight.

The practice of storing water in direct sunlight was only observed in Jordan, where it likely emerged in response to excessive chlorination levels during the early operation of the camp’s water system, which the population, who were unaccustomed to unchlorinated water, found objectionable. Table 3 shows a statistically significant effect of storing water in direct sunlight on lowering household FRC in both Jordan 2014 and Jordan 2015. Table 4 shows that storing water in direct sunlight led to significantly faster FRC decay in both datasets, with the difference being even more pronounced during the hot summer months of the 2014 study. Based on the analysis of these data, we found strong and consistent evidence that storing water in direct sunlight increases FRC decay and compromises the safe water chain.

#### Drawing method.

The method of drawing water for drinking was only assessed in South Sudan and Rwanda because all water users in Jordan drew water by pouring or tap. Table 3 shows that no statistically significant effect was observed between the method of drawing water and household FRC. Table 4 shows that a statistically significant effect was observed in drawing water by pouring or tap with slower FRC decay rates in Rwanda, but not in South Sudan. Based on the analysis of these data, we found some statistically significant evidence, although inconsistent across sites, that drawing water by tap or pouring reduces FRC decay and is protective of the safe water chain, albeit with the caveat that there were only two observations of drawing water by dipping in Rwanda (Figure 2).

#### Water mixing.

Table 3 shows a statistically significant relationship between water mixing and higher household FRC in South Sudan, but no other statistically significant relationships at other sites. Conversely, in Table 4, water mixing in Rwanda is associated with significantly faster FRC decay, but no other statistically significant relationships emerge elsewhere. Based on the analysis of these data, we find mixed and inconclusive results on the effect of water mixing on household FRC and FRC decay.

#### Water transferring.

In Table 3, we observe that transferring water between containers in the home was associated with lower household FRC, which is an effect that was statistically significant in Jordan 2014 and Rwanda, but not in Jordan 2015 and South Sudan. In Table 4, we observe that transferring water between containers in the home was associated with significantly faster FRC decay overall and in South Sudan, Jordan 2015, and Rwanda, but not in Jordan 2014. Based on the analysis of these data, we found fairly consistent evidence, albeit not at every site, that transferring water between containers in the home increases FRC decay and compromises the safe water chain, whereas storing and using water in the same container that was used for collection helps preserve residual chlorine and thus the safe water chain.

#### Water use.

In Table 3, we observe a statistically significant association between reported water use and lower household FRC in Rwanda, whereas there was no significant effect observed at other sites. Similarly, in Table 4, we observe a statistically significant association between water use and faster FRC decay overall and in South Sudan, although it was not significant within the other sites. Based on the analysis of these data, we found some evidence, albeit inconsistent across sites, that active user interactions with stored water in the home were associated with greater FRC decay and lower household FRC.

## DISCUSSION

From the results presented in the preceding section, the two practices that had the clearest effect on household FRC outcomes and FRC decay were 1) storing water in direct sunlight and 2) transferring water between containers during household storage, both of which were found to increase FRC decay, thus reducing protection against recontamination and compromising the safe water chain.

The observed increase in FRC decay rate due to water being stored in direct sunlight is consistent with past research, from which it is known that sunlight degrades the hypochlorite ions that make up FRC into oxygen, chlorite ions, and chloride ions.^35^ Storing water in direct sunlight can also increase water temperature, which can accelerate FRC decay.^36^ Storing water in direct sunlight is fortunately not a common practice in most humanitarian settings. It may have emerged specifically at the Azraq Camp in Jordan in response to excessive FRC levels (i.e., >3 mg/L) at tapstands at the time that the camp was built and the water supply system was brought online in 2014. Storing water in direct sunlight may have arisen as a coping strategy, which later became entrenched, for the population at Azraq, who were unaccustomed to drinking chlorinated water in rural Syria and may have found the taste and odor of excessively chlorinated water objectionable.^34^ Humanitarian WASH teams should be quick to respond if water users are observed storing water in direct sunlight, given the effect it has on reducing FRC protection and compromising the safe water chain. Where this practice is observed, WASH teams should work to better control tapstand FRC levels and intensify community engagement to understand water users’ perceptions of their water supply, especially taste and odor acceptability. Hygiene promotion messaging emphasizing the public health benefits of drinking chlorinated water can also help increase user acceptability of treated water supplies.^40^

Transferring water between containers during household storage was also found to increase FRC decay and compromise the safe water chain. Transferring water between containers may create opportunities for contaminants in the domestic environment to come into contact with the water or container, and it may also represent moments when unhygienic water handling can occur, both of which can drive FRC decay. Increased water safety risk due to transferring drinking water between containers has been previously highlighted by Clasen and Bastable,^26^ who found that transferring water to a second container for storage led to an increase in contamination (although this increase was not statistically significant), as well as by Opryszko et al.,^27^ who suggested that transferring water from transport containers to storage containers in the home introduced another point of potential recontamination. On the other hand, Trevett and Carter found that using separate containers for collection and storage was protective of water quality, albeit in a non-emergency setting.^28^ Although we did not collect data on how many water containers households have, an insufficient number of containers for storing the daily water requirement of a household may oblige people to transfer and consolidate water from multiple vessels to make containers available for further water collection. WASH teams should endeavor to provide a sufficient number of water containers to allow households to collect, store, and use water in the same container (in situations in which the same type of container is used for all of these purposes) without having to transfer between containers. United Nations High Commissioner for Refugees emergency WASH indicators state that ≥70–80% of households should have at least 10 L/person of potable water storage capacity in acute or stabilizing situations.^41^ Ensuring that this indicator is achieved may help reduce pressure on households to transfer water between containers, which can compromise the safe water chain. An additional factor specifically related to FRC decay that was not considered in previous studies of microbiological contamination is the effect of agitation on chlorine decay; transferring water between containers could agitate water, thereby increasing the volatilization of FRC and resuspending settled organics that may react with the FRC.

For the three key hygienic water handling practices recommended by humanitarian guidelines—ensuring container cleanliness, covering containers, and drawing water by pouring or tap^10^—findings with respect to protective effects were inconclusive for the former two, whereas some inconsistent evidence was found for the protective effect of the latter. This is in keeping with the literature, which presents a similarly mixed picture regarding the protective effects of hygienic water handling practices vis-à-vis microbiological recontamination and waterborne illnesses (we looked at FRC in this study, but all these outcomes lie along the same causal pathway). Shultz et al.^4^ found that storing water in a sealed or covered container was associated with significantly lower rates of cholera during an outbreak in the Kakuma Refugee Camp in Kenya. Swerdlow et al.,^6^ in another study, found that higher levels of cholera were associated with users putting their hands into water containers in the Nyamithuthu Refugee Camp in Malawi. A 2004 meta-analysis by Wright et al.^23^ found that the use of covered containers was significantly related to the degree of fecal coliform contamination, at least when source water had low initial coliform levels. However, several other studies have found that container covering did not significantly affect the presence or degree of microbiological contamination.^19^^–^^21^^,^^25^^,^^28^ Similarly, drawing water from a tap can prevent water users from dipping unclean vessels into the water, and this has been found, in some cases, to reduce the magnitude of microbiological contamination^26^ or the likelihood of such contamination occurring.^41^ However, another study did not detect a significant protective effect of drawing water by tap.^19^ Potential reasons that the evidence was inconclusive or inconsistent are discussed in the next section.

For water mixing, we found mixed and inconclusive results on the effect it had on household FRC outcomes and FRC decay. This may have been because, where water mixing was reported, it was unknown whether the original unit of water was mixed with older water in the home (with presumably lower FRC) or with newer water brought from the tapstand (with presumably higher FRC, but that too can fluctuate). For reported water use, we found some inconsistent evidence across sites that active user interaction with stored water in the home was associated with faster FRC decay and lower household FRC. We would presume that user interactions provide an opportunity for recontamination to occur, but this may not have always been the case. These ambiguities are discussed in the next section.

### Study limitations.

#### Data reliability.

The inconclusive or limited evidence we found on the protective effects of the recommended hygienic water handling practices may be partially explained by how these data were collected. Container covering was observed via spot-check by surveyors, and the drawing method was based on respondent self-reports during household follow-up visits. Spot-check observations do not document what happens in the hours before the observation occurs. For example, a container may have been left uncovered for several hours before being covered again before the household water handling survey. In addition, although a preliminary analysis did not find a significant effect of container type because of imbalanced data, we are aware that some of the containers used have narrow apertures. Numerous studies have proposed or investigated improved containers with narrow apertures to provide a physical barrier to recontamination.^5^^,^^18^^,^^29^^,^^30^ Thus, some of these containers still have a physical barrier against recontamination, even when uncovered. An additional data limitation is that respondent self-report may not capture that different people in the household could have different water drawing practices than the survey respondent. Respondent self-reporting may also be subject to recall or courtesy bias. Container cleanliness was documented through a visual assessment of the inside and outside of water collection containers by surveyors based on a common typology of containers at each site, and subjective visual inspection may fail to consistently characterize the degree of cleanliness observed by different surveyors or even by the same surveyor. These factors could have weakened the reliability of the data on these key hygienic water handling practices, which in turn could obscure effects between their binary categories. Furthermore, a visual assessment of cleanliness may not identify biofilm growth, which could consume residual chlorine,^42^ in a container that appears clean, and conversely, staining, weathering, and bleaching from sunlight may make a clean container look unclean; thus, there is a possibility of misclassification in both directions using visual assessments. Methods that can overcome these limitations and should be considered for future studies include structured observations,^43^^,^^44^ repeated observation spot*-*checks,^45^ allowing for multiple categorizations,^46^^,^^47^ and novel behavioral sensing devices.^48^

With regards to container cleanliness specifically, there may be additional concerns about what is being measured. Keeping containers clean is important for preventing microbiological growth because this can lead to outbreaks of waterborne illness, as was observed at the Abou Shouk Refugee Settlement in Sudan or the Kitgum IDP Settlement in Uganda.^7^^,^^49^ In both cases, container cleaning and disinfection helped end outbreaks of diarrheal diseases. However, visual inspections of container cleanliness may be insufficient. A study in rural Cambodia by Benwic et al.^25^ found that households that stored water in vessels that appeared dirty on the outside were more likely to be contaminated than vessels that did not; however, they did not find a significant difference based on the cleanliness inside the container or based on visual inspection of the inside and outside of the container (as was the case with the dataset used in this study). An alternative indicator of container cleanliness may instead be a history of how recently the container was cleaned, but the same study in Cambodia by Benwic et al.^25^ did not find any significant difference between the presence and absence of microbiological contamination based on either the frequency of cleaning or whether treated water was used for cleaning. Additionally, even when cleaning is effective, it must be sustained, as was seen during the outbreak at the Kitgum IDP settlement. At this site, shock disinfection initially reduced *Escherichia coli* concentrations, but within 3–5 days, bacteria were once again present—sometimes at greater concentrations than before the shock chlorination campaign.^49^ An effective approach to maintaining container cleanliness may be to regularly disinfect containers, as was demonstrated by two recent studies conducted in peri-urban settlements, which found that microbiological contamination and FRC loss during storage could be significantly reduced by using professionally disinfected containers every time water is collected.^33^^,^^42^ Such an approach could also be considered for ensuring safe storage in refugee and IDP settings. Another more sophisticated approach to measuring container cleanliness may be to directly measure biological activity in biofilms in containers using adenosine triphosphate because biofilm activity provides an indication of the magnitude of microbiological contamination.^50^

Another factor that could obscure the protective effect of the recommended hygienic water handling practices is that the observed absence of these practices does not necessarily entail that contamination will occur—only that the opportunity for contamination exists. Keeping a container uncovered does not guarantee that contaminating material will fall in; a visually unclean container may not exert a chlorine demand; and dipping a utensil to draw water may not introduce contamination if the utensil itself is clean. This stands in contrast to storing water in direct sunlight, which necessarily drives chlorine decay as photonic degradation of FRC is impossible to avoid if the conditions are right. This could explain the relative strength of the observed effect of storing water in direct sunlight compared with the inconclusive or mixed effects seen for hygienic water handling practices.

#### Imbalanced data.

Another factor that may have contributed to the inconclusive or mixed effects observed for some hygienic water handling practices is that the dataset used for this secondary analysis was not specifically designed for this purpose. These data were collected for the primary purpose of modeling post-distribution FRC decay.^34^ Water handling practices were not equally distributed at each site, resulting in imbalanced data on several variables (Figure 2). The sample size at each site may have been inadequately powered to evaluate FRC effects associated with each behavior. Stronger conclusions may be possible with a larger and more balanced behavioral dataset.

#### Multiplicity of tests.

The multiplicity of tests across the two analytical approaches taken indicates a high cumulative likelihood of a type I error.

### Implications for practitioners.

Although all of the key hygienic water handling practices recommended by humanitarian guidelines were not found to consistently reduce FRC decay in the present analysis with this dataset, they are still likely effective measures for promoting water safety by virtue of the fact that they provide physical barriers to the ingress of waterborne pathogens.^26^^,^^29^^,^^47^ These should, therefore, continue to be promoted as essential safe water chain measures in humanitarian settings.

The complexity of the relationships between water handling and residual chlorine protection has implications for water treatment in humanitarian settings, as well. To support water treatment operations, FRC decay modeling is used to generate site-specific and evidence-based water chlorination targets that ensure that water remains safe to drink all the way up to the point-of-consumption in refugee and IDP settlements.^8^^,^^34^^,^^37^^,^^39^ A major challenge when developing models of post-distribution FRC decay is the high degree of uncertainty in FRC decay rates. Water samples collected on the same day at the same site exhibit considerably different decay behaviors because of highly variable environmental, water quality, behavioral, and other factors during the post-distribution period. Attempting to deterministically characterize the effect of every single water handling behavior on post-distribution FRC decay or any other factor would be challenging, if not outright impossible. Given the complexity characterized by this study, it may be preferable to prioritize FRC decay modeling approaches that can handle uncertainty in post-distribution decay in a probabilistic manner. Probabilistic models can be used to produce site-specific and evidence-based water chlorination targets not by outputting fixed targets but by presenting the risk of having insufficient FRC after a specified duration of household storage in a manner that integrates the complex effects of environmental, water quality, behavioral, and other factors that drive chlorine decay.

## CONCLUSION

Ensuring hygienic water handling practices, such as covering containers, drawing water via tap or pouring, and keeping containers clean, as well as maintaining residual chlorine protection throughout the entire post-distribution period of collection, transport, and household storage is critical for preventing pathogenic recontamination and preserving the safe water chain in humanitarian settings. In this secondary analysis study, we explored how water handling practices affect household FRC and post-distribution FRC decay. We found that the two practices that had the clearest effect on residual chlorine protection were storing water in direct sunlight and transferring water between containers during household storage, both of which increased FRC decay and compromised the safe water chain. Humanitarian WASH teams should be quick to respond if water users are observed storing water in direct sunlight, given the effect it has on driving off FRC protection and compromising the safe water chain. Where this practice is observed, WASH teams should work to better control tapstand FRC levels in an acceptable range and intensify community engagement to understand water users’ perceptions of their water supply, especially taste and odor acceptability. Hygiene promotion activities should also be undertaken to encourage user acceptability of chlorinated water supplies and dissuade water users from storing water in direct sunlight if this coping strategy is observed. WASH teams should endeavor to provide sufficient numbers of water containers to allow households to collect, store, and use water in the same container without having to transfer between containers because water transferring presents opportunities for recontamination to occur. Providing sufficient numbers of containers can also enable regular cleaning and disinfection of water containers. With respect to the three key hygienic water handling practices, we found mixed and inconclusive evidence on the protective effect of container cleanliness and container covering and some limited evidence on the protective effect of drawing water by tap or pouring. Hygienic water handling behaviors provide an important physical barrier against the introduction of waterborne pathogens and should always be promoted in humanitarian settings. Both residual chlorine protection and hygienic water handling practices are essential chemical and physical barriers to pathogenic recontamination of treated water drinking water and are critical for protecting the safe water chain in humanitarian settings.

## Supplemental Materials

10.4269/ajtmh.24-0221 Supplemental Materials
