# Supplementary material for: Protecting the Safe Water Chain in Refugee Camps: An Exploratory Study of Water Handling Practices, Chlorine Decay, and Household Water Safety in South Sudan, Jordan, and Rwanda
Source: Am J Trop Med Hyg. 2024 Dec 3;112(2):451–61. doi: 10.4269/ajtmh.24-0221 (PMC11803672; doi:10.4269/ajtmh.24-0221)
Supplement: Supplemental Materials [file tpmd240221.SD1.pdf]

SUPPLEMENTARY MATERIALS

for

Protecting the safe water chain in refugee camps: An exploratory study of water handling practices, chlorine decay, and household water safety in South Sudan, Jordan, and Rwanda

Syed Imran Ali[1,2,3*], Michael De Santi[1,4], Georges Monette[5], Usman T. Khan[1,4], Jean-Francois Fesselet[2], and James Orbinski[1]

[1] Dahdaleh Institute for Global Health Research, York University, Toronto, Canada

[2] Médecins Sans Frontières, Public Health Department, Amsterdam, The Netherlands

[3] Blum Center for Developing Economies, University of California, Berkeley, USA

[4] Civil Engineering, Lassonde School of Engineering, York University, Toronto Canada

[5] Department of Mathematics and Statistics, York University, Toronto Canada

** Corresponding author contact: siali@yorku.ca, Dahdaleh Institute for Global Health Research, Suite 2150, Victor Dahdaleh Building, York University, 4700 Keele St, Toronto, Ontario, Canada, M3J 1P3, 416 736 2100 x 34447*

Number of supplementary files: 3 (1 table, 2 figures)

Table S1: Photographs from field sites illustrating the categories for container cleanliness, covering, drawing method, and storage in direct sunlight. (Photo credits: Syed Imran Ali).

**Site**	**Behavior Demonstrated**	**Image**
South Sudan	Clean, Covered	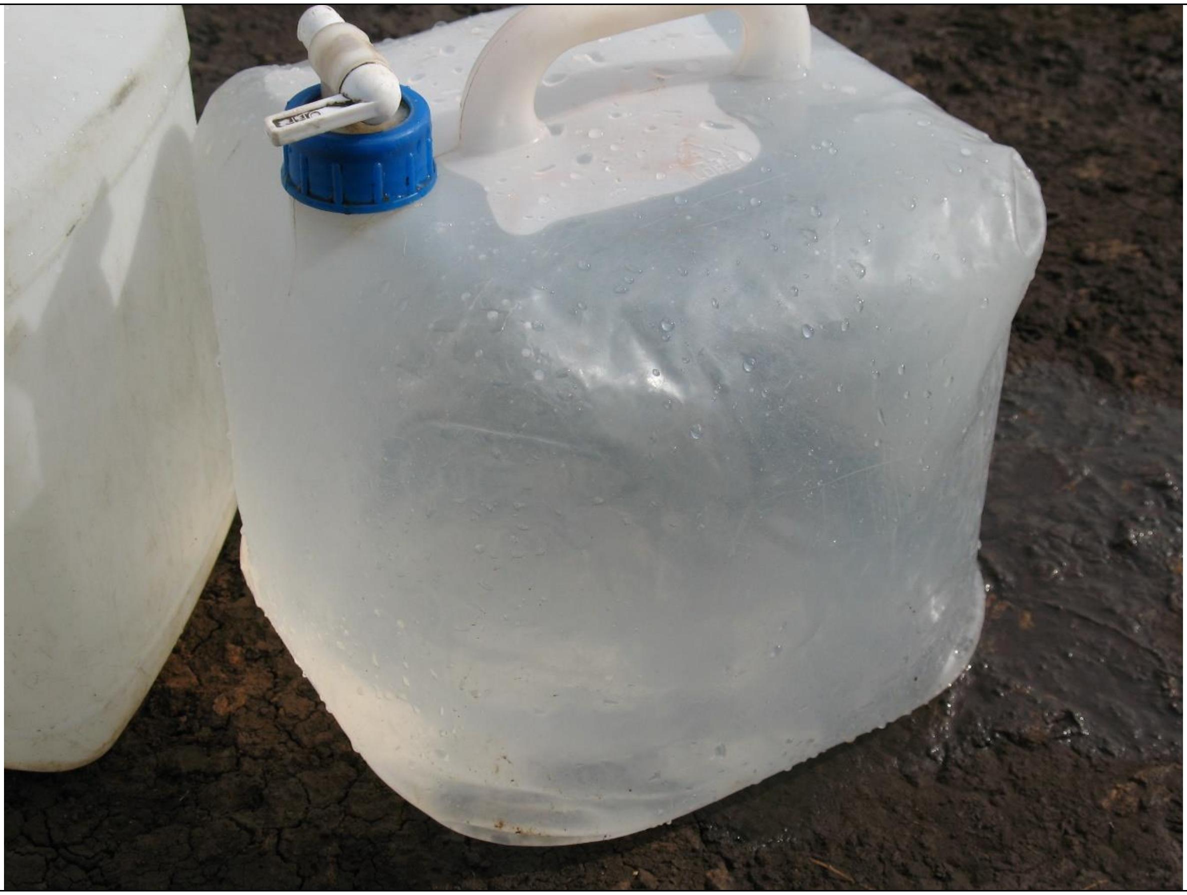

South Sudan	Clean, Uncovered	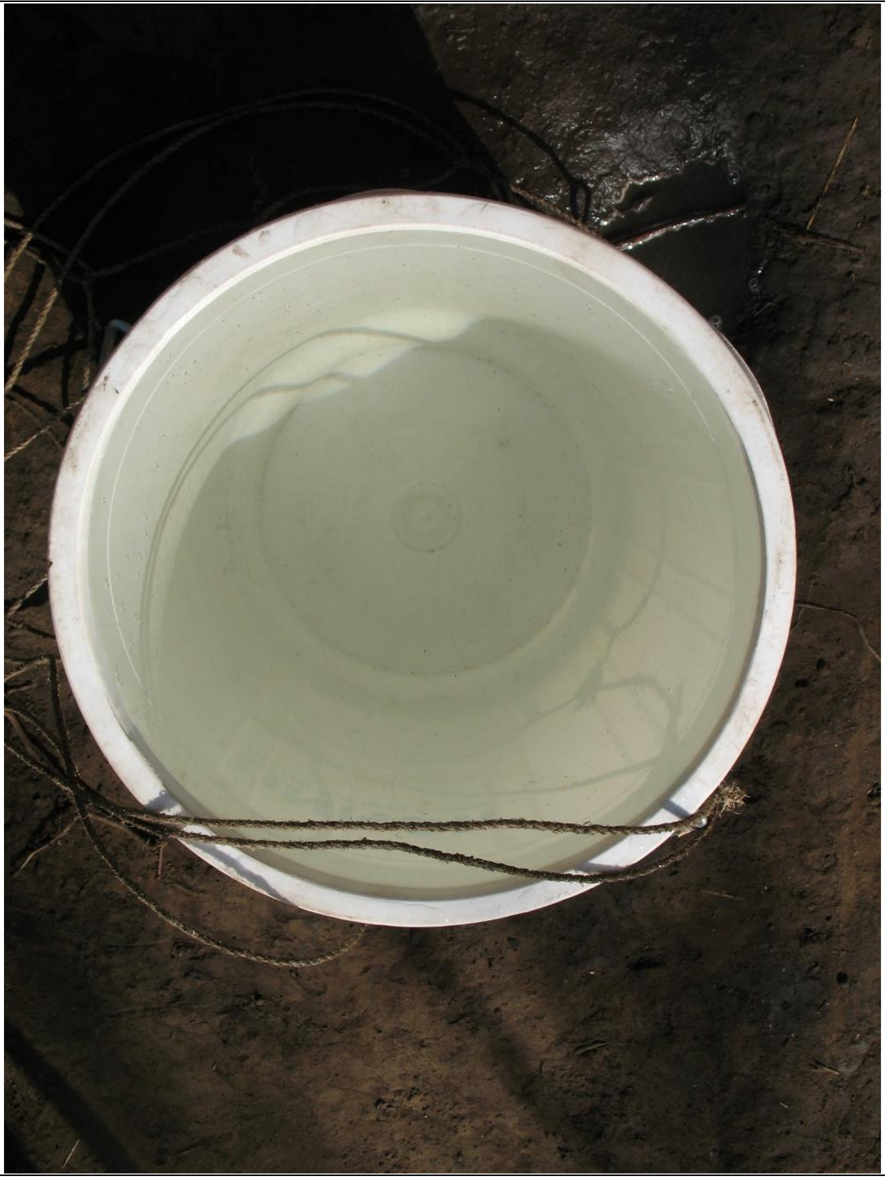	

Site	Behavior Demonstrated	Image
South Sudan	Covered	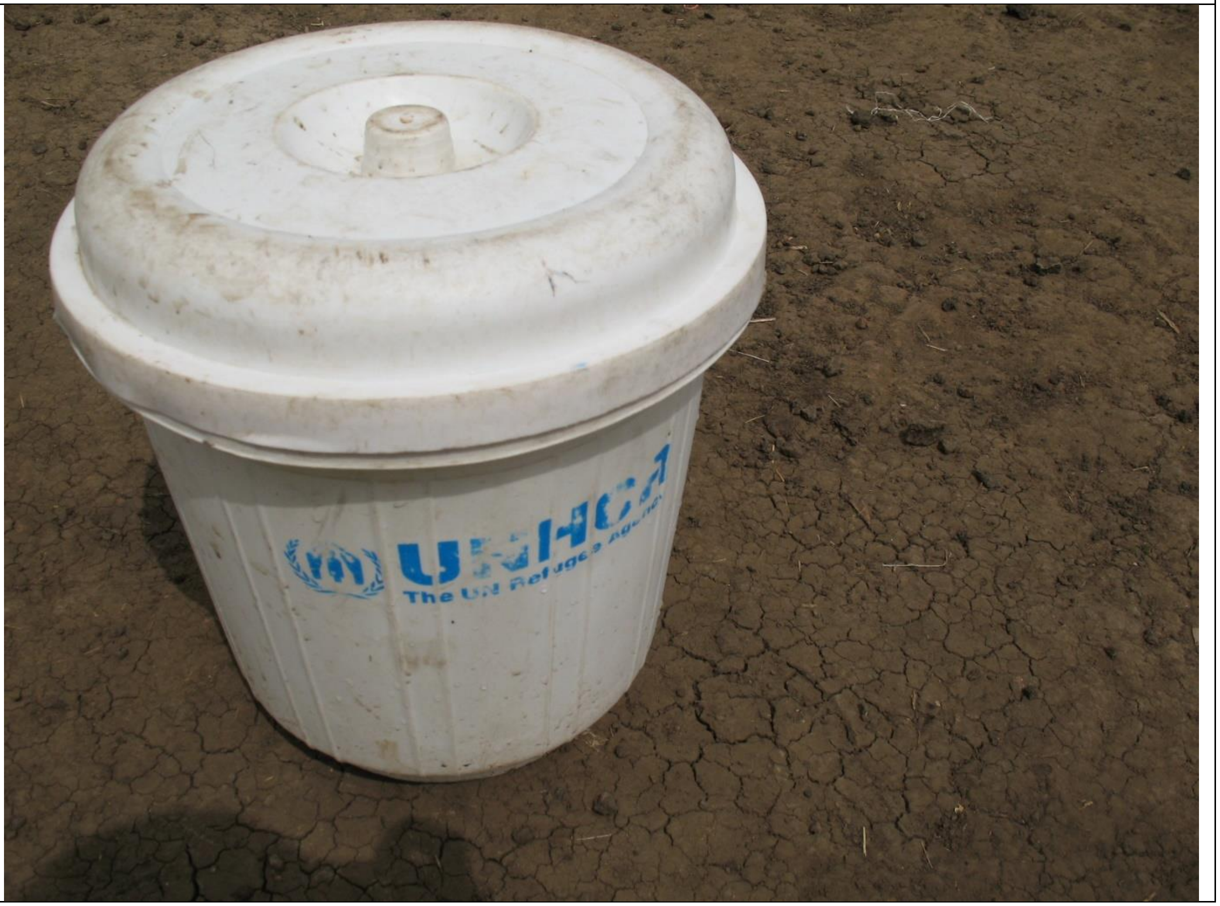

Site	Behavior Demonstrated	Image
South Sudan	Uncovered	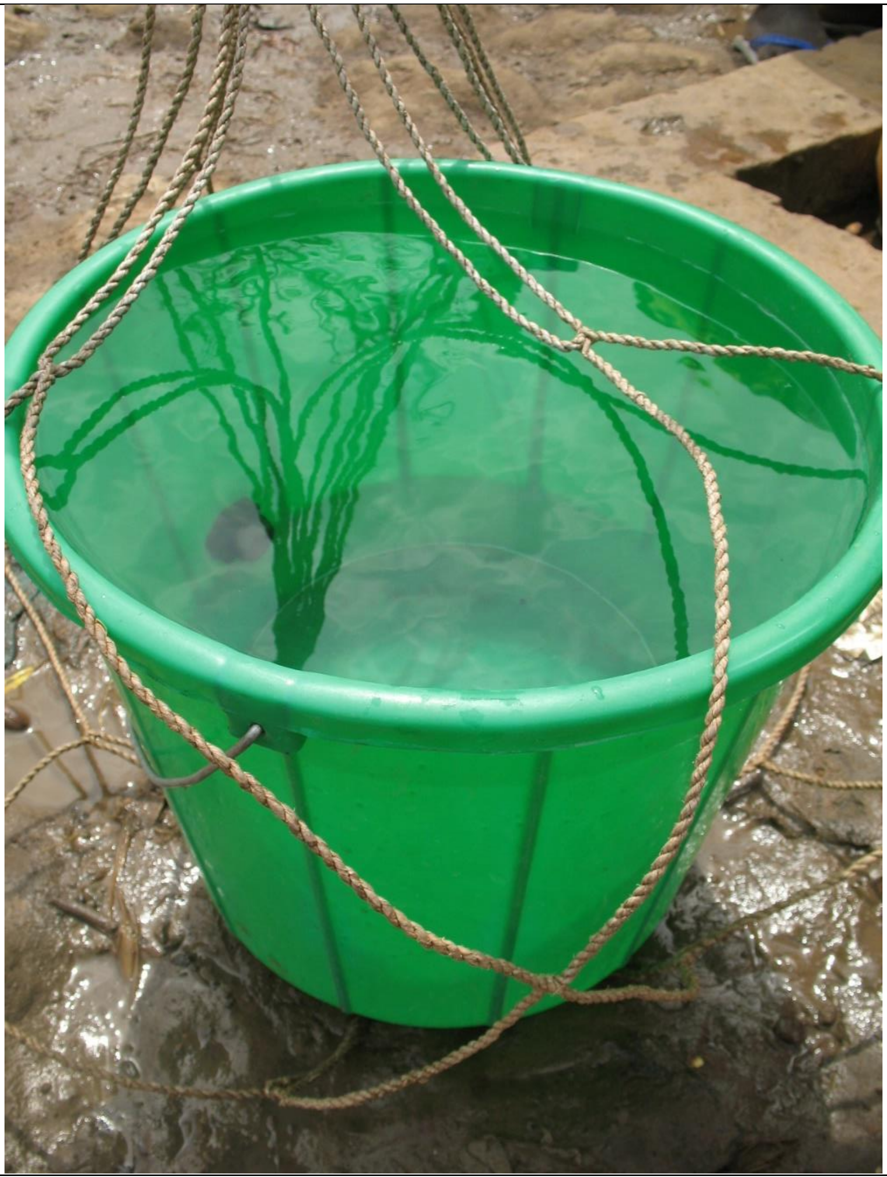

Site	Behavior Demonstrated	Image
South Sudan	Unclean	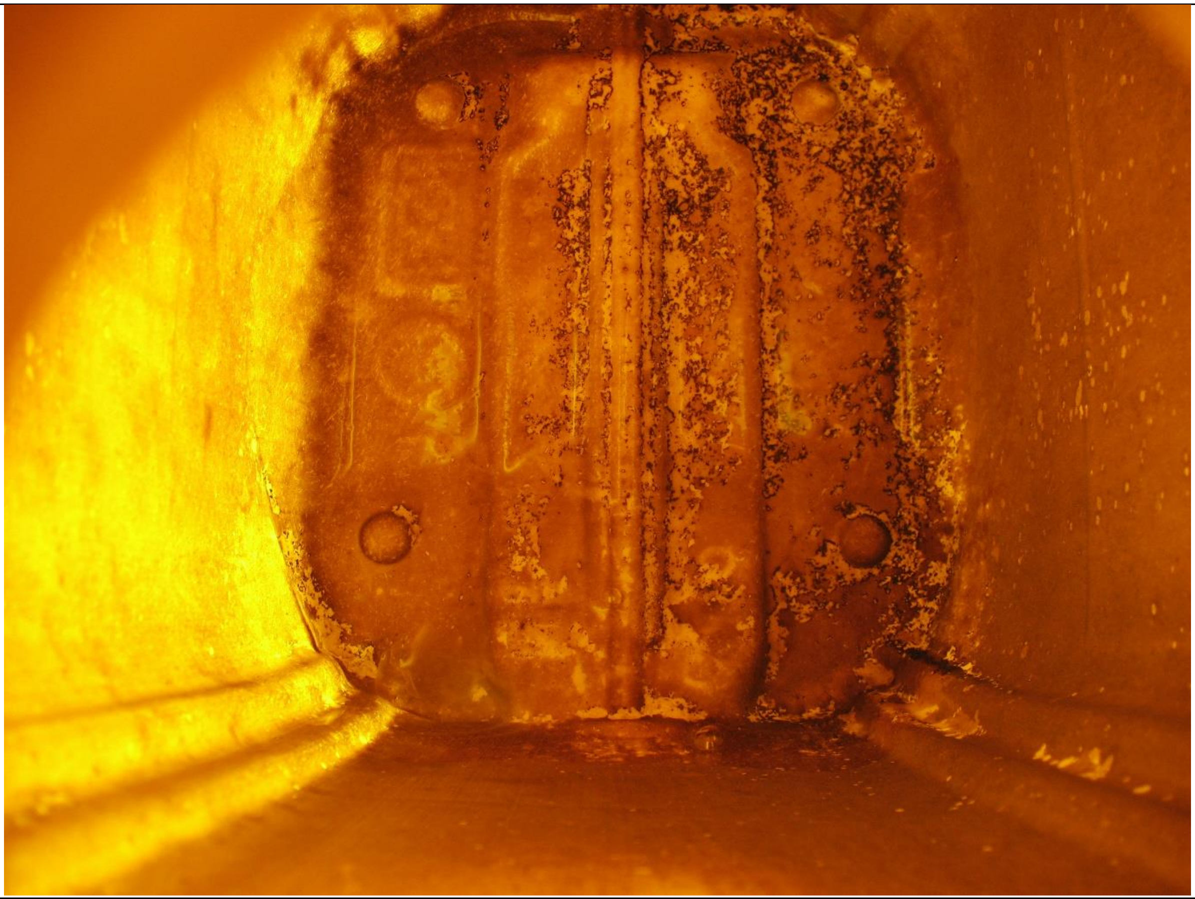

South Sudan	Uncovered, unclean	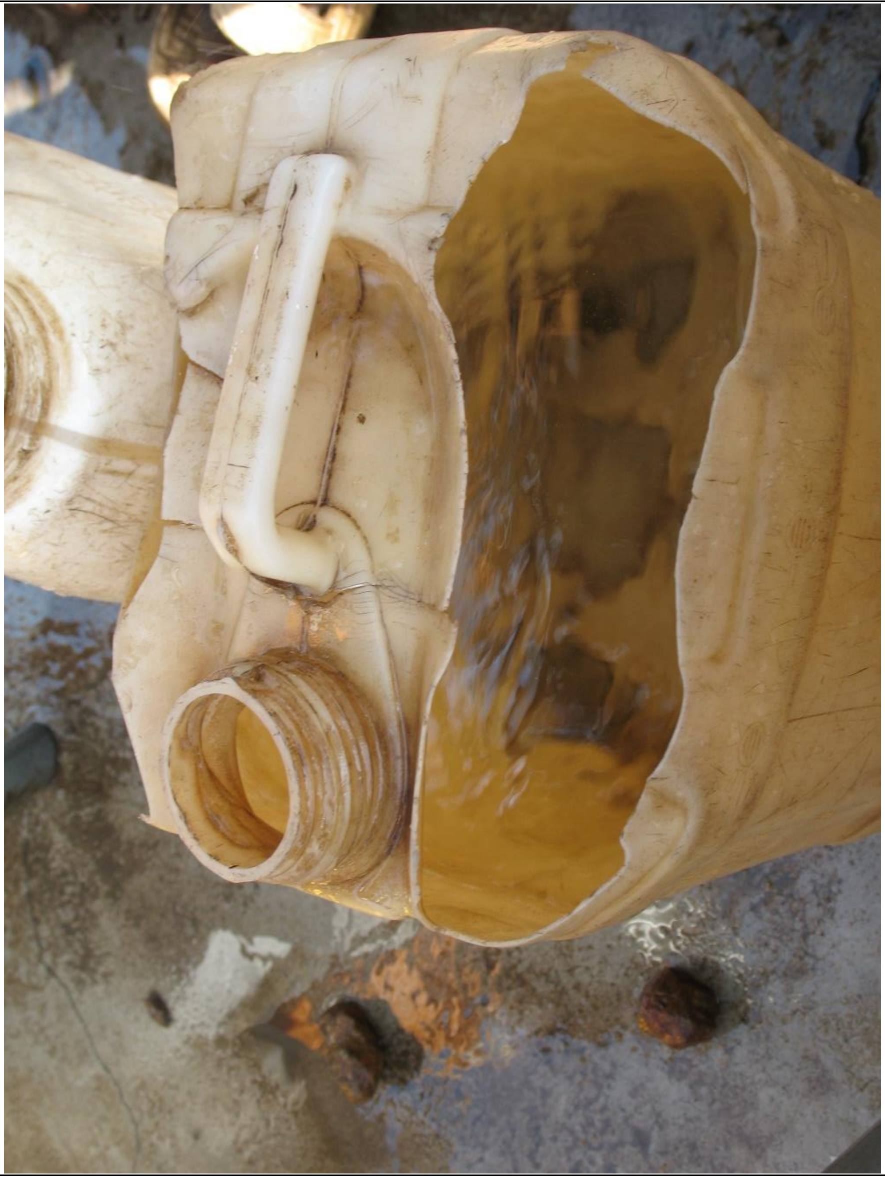	

Site	Behavior Demonstrated	Image
South Sudan	Drawing Method - Dip	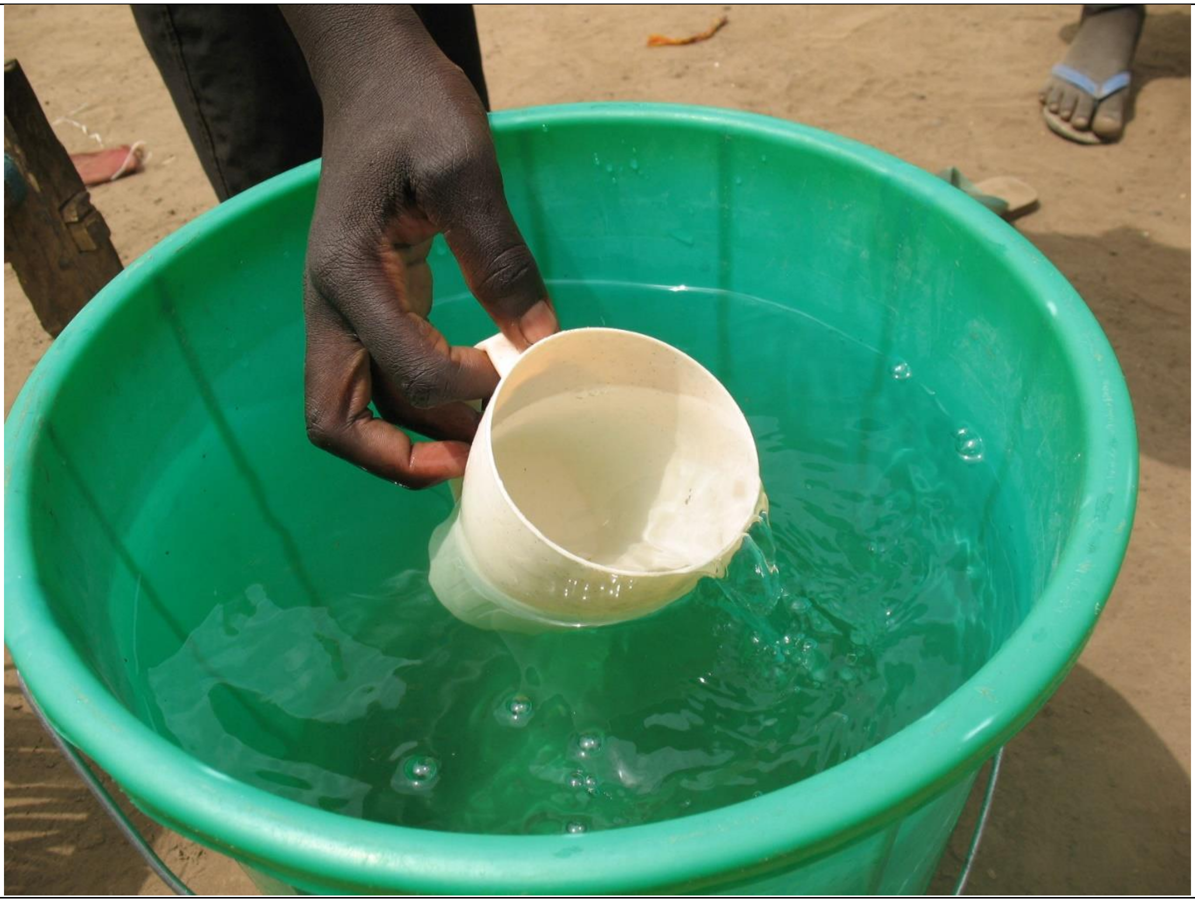

Site	Behavior Demonstrated	Image
South Sudan	Drawing Method - Pour	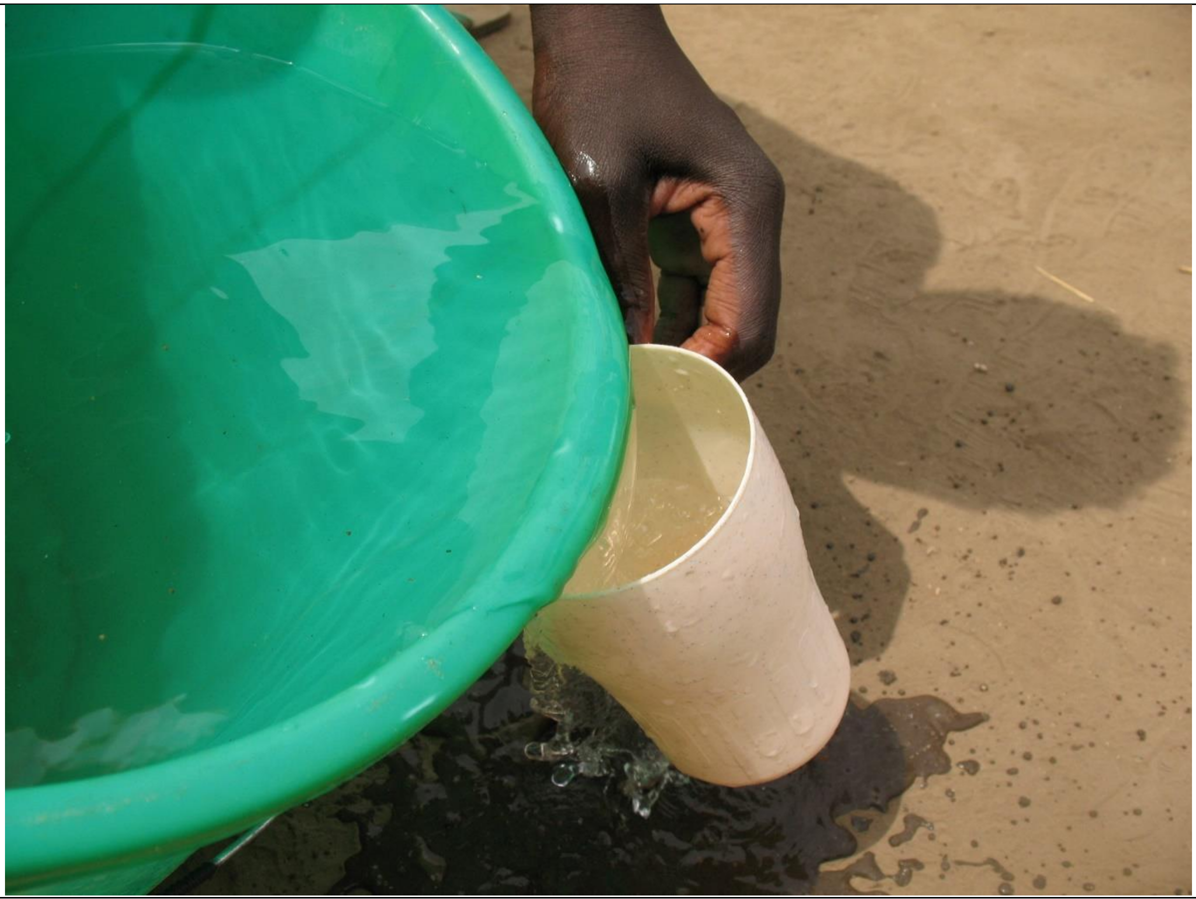

Site	Behavior Demonstrated	Image
South Sudan	Drawing Method - Tap	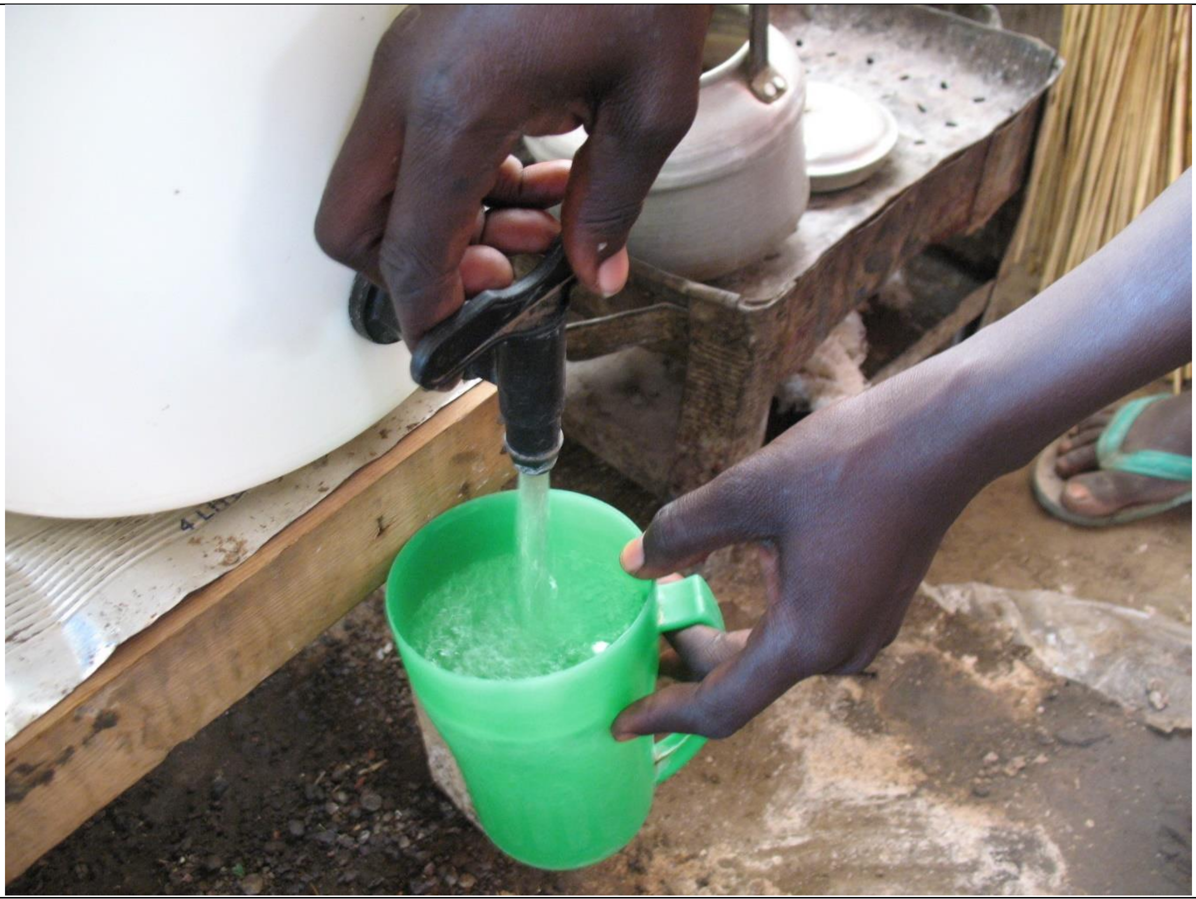

Jordan (2014)	Clean, Covered	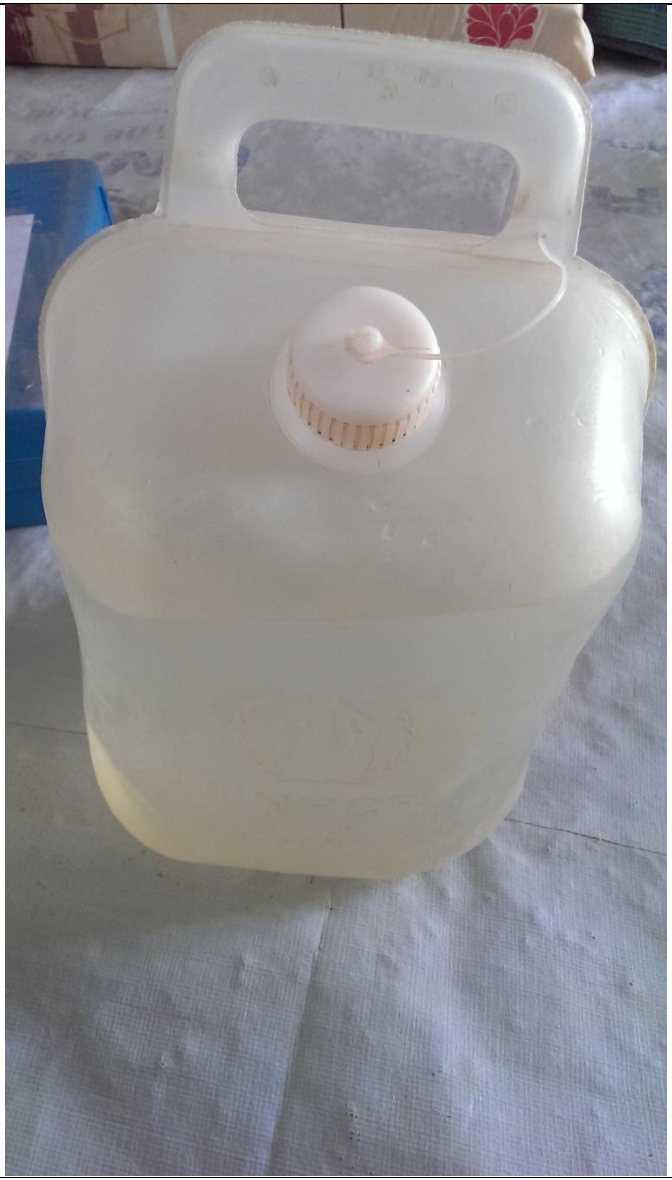	

Jordan (2014)	Covered	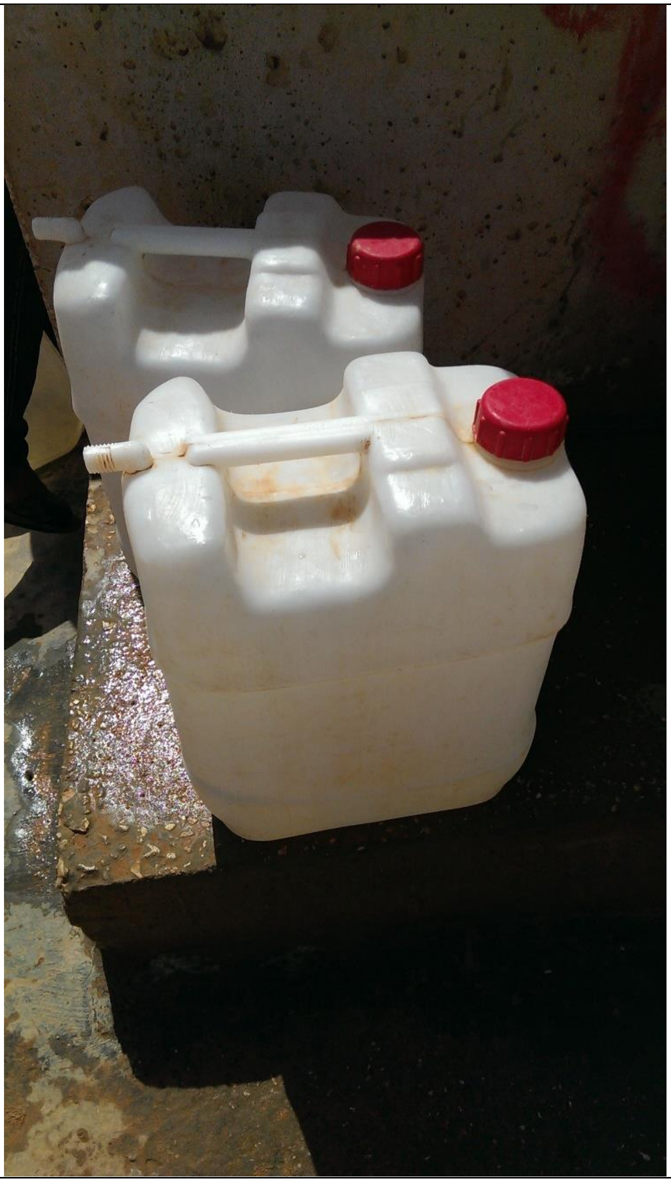	

Jordan (2014)	Uncovered	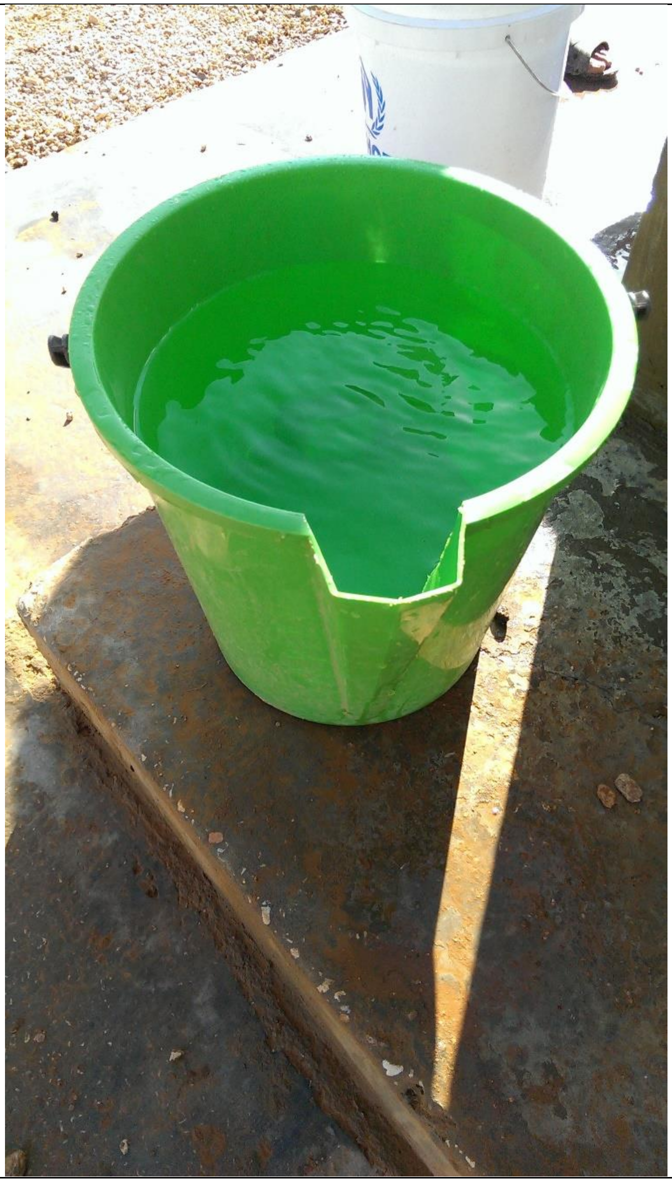	

Site	Behavior Demonstrated	Image
Rwanda	Uncovered	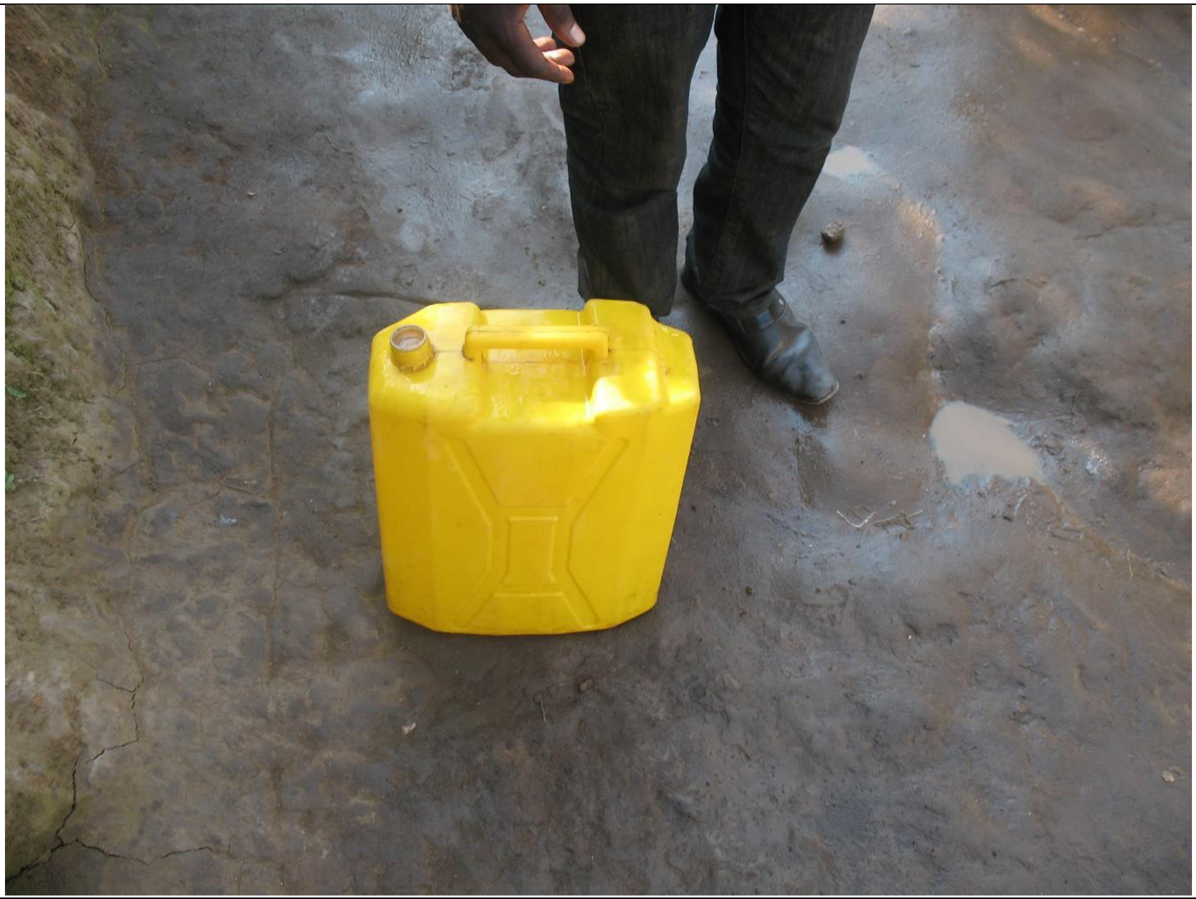

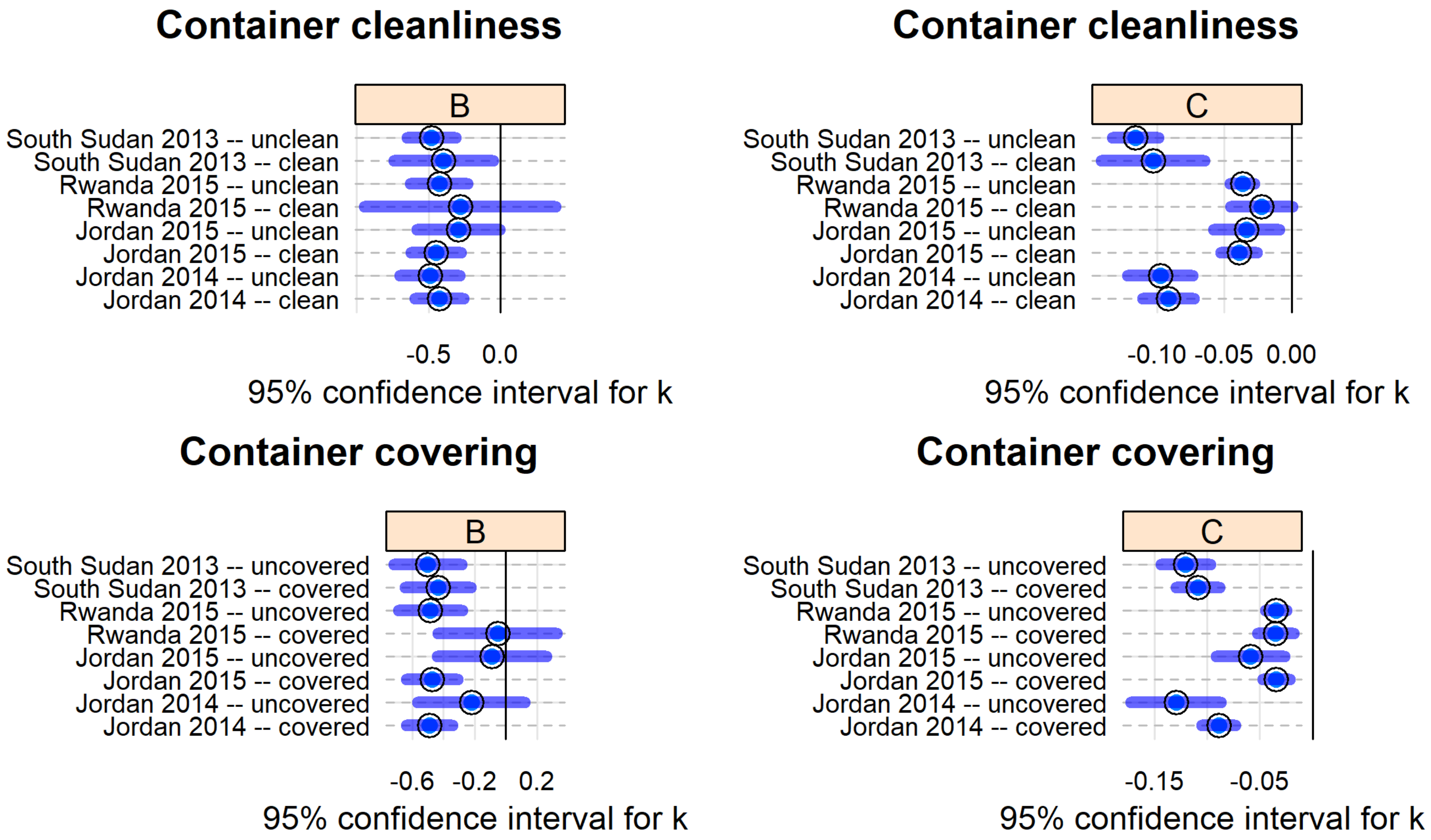

Figure S1: Estimated decay rates, *k*, with confidence intervals from the linear mixed effects model for container cleanliness and container covering in phases B (collection and transport) and C (storage and use).

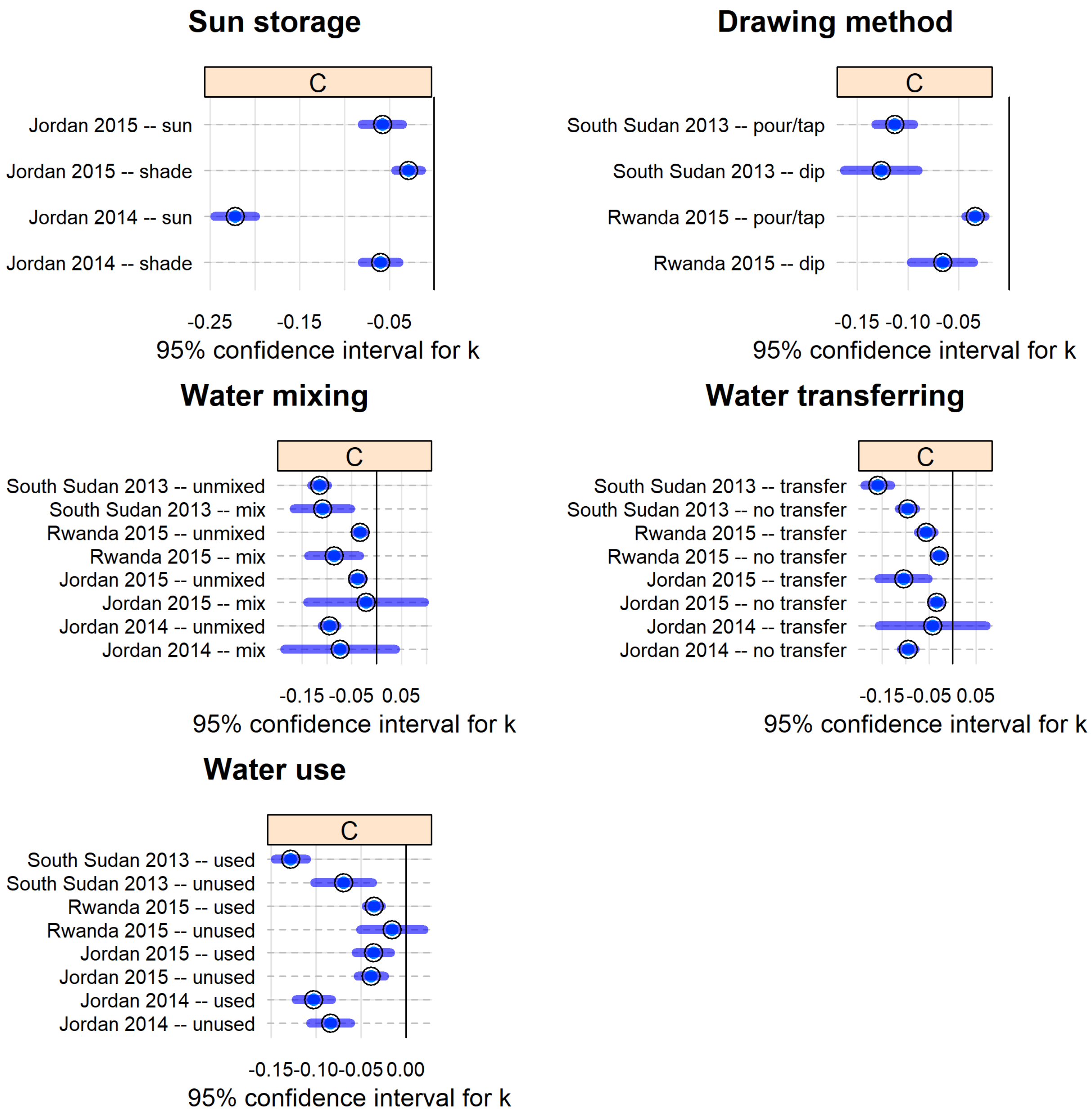

Figure S2: Estimated decay rates, *k*, with confidence intervals from the linear mixed effects model for sun storage, drawing method, water mixing, water transferring, and water during phase C (storage and use).
